# Associations of living arrangements with symptoms and functioning in schizophrenia

**DOI:** 10.1186/s12888-021-03488-5

**Published:** 2021-10-11

**Authors:** Mei San Ang, Gurpreet Rekhi, Jimmy Lee

**Affiliations:** 1 Research Division, Institute of Mental Health, Singapore, Singapore; 2 North Region & Department of Psychosis, Institute of Mental Health, Singapore, Singapore; 3 Lee Kong Chian School of Medicine, Nanyang Technological University, Singapore, Singapore

**Keywords:** Schizophrenia, Living arrangements, Accommodation, Symptomatic remission, Functioning

## Abstract

**Background:**

Living arrangements and accommodation are closely related, but no study had concurrently investigated their associations with outcomes in schizophrenia. This study seeks to describe and compare socio-demographic, clinical and functioning profiles of people with schizophrenia in different living arrangements and accommodation, and to examine the associations of living arrangements and accommodation with symptomatic remission and functioning.

**Methods:**

Community dwelling outpatients with schizophrenia (*n* = 276) were inquired on living arrangements, accommodation, socio-demographics and assessed on the Positive and Negative Syndrome Scale (PANSS) and the Social and Occupational Functioning Scale (SOFAS). Socio-demographics, symptoms and functioning of outpatients in different living arrangements and accommodation were compared. Symptomatic remission was investigated using logistic regression with living arrangements, socio-demographics and clinical variables as independent variables. Functioning was investigated using multiple regression with the same set of independent variables and the addition of PANSS factors. The same analyses were conducted with accommodation as independent variable.

**Results:**

185 (67.03%) participants lived with family and 195 (70.65%) participants lived in owned accommodation. People living with their spouses had significantly higher SOFAS, lower PANSS Total and PANSS Positive than people living with family, independently, or in rehabilitation centres. They also had lower PANSS Negative than people living with family and a higher likelihood to have achieved symptomatic remission. Types of accommodation was not associated with symptoms, symptomatic remission, and functioning.

**Conclusion:**

Living arrangements, but not types of accommodation, were associated with symptoms and functioning in schizophrenia. Family education and support is important to help maintain a conducive environment for people with schizophrenia. People living independently may need more support.

## Introduction

Living arrangement, which refers to where a person lives and who the person lives with, is an important base for everyday living where daily activities and social interaction take place. Living arrangements and social network were associated with symptoms and functioning in people with schizophrenia [1–3]. Social relationships within a living arrangement are important in a person’s social network. People in the same living arrangement are often the confidants for people with schizophrenia [4]. Social relationships help to meet patients’ needs in recovery process, such as social approval and integration, material support, problem solving and symptom monitoring [5]. People with schizophrenia often rely on help from relatives or friends with their difficulties in areas such as psychotic symptoms, finances and companionship [6]. Unfortunately, people with schizophrenia have smaller network sizes made up of a higher proportion of family and fewer friends and close relationships [7–9]. Coupled with limited personal resources, it’s unsurprising that approximately half of patients with schizophrenia stayed with their family or their loved ones [10].

Accommodation, which refers to the place a person lives in, is one of the needs in outpatients with schizophrenia [6]. Types of accommodation affected stability of stay, activities that could be conducted, and the establishment of supportive social relationships, all of which are beneficial to mental health [11]. Adequacy of living situation impacts on maladaptive behaviours, functioning and quality of life [12–14]. Living in supervised residential settings were associated with lower living skills and functional abilities [15–17]. People with schizophrenia discharged to boarding houses were also more likely to be re-hospitalized [18].

Studies that directly inquire the association of living arrangements and accommodation with symptoms and functioning are scarce [15]. Further, conflicting findings were observed in the literature. Salokangas (1997) suggested that people living with their spouses had significantly better functioning than all other groups but Tsai et al. (2011) reported that patients living alone independently had significantly better social and role functioning than all other groups. Findings were also not readily comparable. Salokangas (1997) showed that people with schizophrenia living with family had fewer negative symptoms than people living with a spouse or people living independently, while Tsai et al. (2011) showed that institutionalized people had more severe negative symptoms than people living with someone or living alone independently.

Different cultures and societal expectations may have influences on living arrangements [19, 20]. Living arrangements of young people were significantly different in European, Western and East Asian countries [19, 21]. Factors such as sex, race, cultural norms and expectation, personal and family resources, close ties with family, national economic situations, and availability of affordable housing were related to independent living [22, 23]. The interaction of these factors made comparison of findings on living arrangements across studies a challenge. More studies investigating the impact of living arrangements on outcomes such as symptoms severity and functioning are needed to fill the gap.

In studies investigating accommodation in people with mental illness, a diagnosis of schizophrenia appeared to confound the results [24]. Not only very few studies were found in a literature review on supported housing, the diverse models of supported housing investigated also made comparison difficult [15]. No study has examined living arrangements and accommodation concurrently, which could potentially unravel the relative association of living arrangements and accommodation with symptomatic and functional outcomes in schizophrenia. This study aimed to (i) compare the differences in socio-demographic, clinical and functioning profiles of community dwelling outpatients with schizophrenia in different living arrangements and accommodation, and (ii) investigate the associations of living arrangements and accommodation with symptomatic remission and functioning, respectively.

## Methods

### Study setting and participants

Community dwelling outpatients with schizophrenia (*n* = 276) were recruited from outpatient clinics in the Institute of Mental Health; 274 completed all clinical assessments. The Structured Clinical Interview for DSM-IV-TR Axis I Disorder-Patient Edition (SCID-I/P) [25] was used to ascertain the diagnosis of schizophrenia. Patients with schizophrenia aged 21–65 years who were able to speak English and provide written informed consent were eligible. Current alcohol or substance use disorder, history of brain injuries, neurological disorder or intellectual disability were exclusion criteria. Written informed consent was obtained from all participants prior to data collection. Ethics approval was obtained from the National Health Group’s Domain Specific Review Board. Data were collected from August 2014 to December 2017.

### Assessments

#### Socio-demographic information was reported by participants

The Positive and Negative Syndrome Scale (PANSS) [26] was used to assess severity of symptoms. The PANSS has 30 items measuring symptoms of schizophrenia rated from “1: Absent” to “7: Extreme”. Items were explained by five underlying factors: positive symptoms, negative symptoms, excitement, depression and cognition/disorganization [27, 28]. With reference to factors suggested by a local validation study [27], factor scores were computed using summation method. PANSS items P1, P3, P6 and G9 constituted PANSS Positive; items N2, N3, N4, N6 and G7 constituted PANSS Negative; items P4, P7 and G14 constituted PANSS Excitement; items G2, G3 and G6 constituted PANSS Depression; and items G10 and G12 constituted PANSS Cognition. Cross-sectional symptomatic remission status was computed according to the criteria proposed by the Remission in Schizophrenia Working Group [29].

The Social and Occupational Functioning Scale (SOFAS) [30] was used to measure functioning. The SOFAS measures social and occupational functioning with a single rating ranging 0–100. Functioning was rated in reference to ten 10-point deciles which describe ten ranges of low to high functioning, with higher scores denoting better functioning.

Clinical scales were rated by 3 raters who had a minimum of 2 years’ experience rating psychiatric symptoms. Intra-class correlation for PANSS established prior to the study was adequate (> 0.80). To ensure adequate consensus in ratings, cases were discussed bi-monthly.

### Coding of variables

Adapting from the categorization of living arrangements used in previous studies (e.g., Salokangas, 1996), living arrangements were coded into four categories: (i) living with spouse: patient formed a new family; (ii) living with family: living with original nucleus family, i.e. parent(s) and/or sibling, or someone in their extended family; (iii) living independently: living with landlord, tenants, roommates, or friends; (iv) living in a rehabilitation centre. Accommodations were categorized into two categories: (i) owned accommodation: public or private housing; (ii) rental accommodation/rehabilitation centres. Rental accommodation and rehabilitation centres were split into two categories to be compared with owned accommodation to scrutinize the results.

Number of illness exacerbation represented the total number of hospitalization and illness exacerbation recorded in medical records. Because of skewed distribution, this variable was recoded by quantiles, ranging 1–5 with 5 indicating the highest quantile of illness exacerbations.

### Statistical analyses

Socio-demographic and clinical characteristics of the participants were summarized using descriptive statistics. Univariate analyses were first conducted to compare the differences on socio-demographic and clinical characteristics of participants in different living arrangements and accommodation. For example, Chi-square test was used to explore the relationship between categorical variables. T-test was used to compare differences of continuous variables by accommodation (two categories), while one-way analysis of variance (ANOVA) was employed to compare differences on continuous variables by living arrangements and accommodation (three categories); Bonferroni tests were used for multiple comparisons. When the assumption of homogeneity of variances was violated, Brown-Forsythe test was used for Omnibus ANOVA and Games-Howell tests for multiple comparisons. For ordinal variables or continuous variables that were non-normal, Mann-Whitney U test was used for comparison between two groups and Kruskal-Wallis H test for comparisons more than two groups. Dunn-Bonferroni tests were used for multiple comparisons if Kruskal-Wallis H test indicated significant difference.

To investigate the impact of living arrangements on symptomatic remission, logistic regression was conducted with symptomatic remission as dependent variable, and living arrangements, socio-demographic (sex, age, ethnicity, years of education) and clinical variables (age of onset of psychotic symptoms, illness exacerbation, physical comorbidity, antipsychotic dose) as independent variables. Multiple regression was employed to examine the impact of living arrangements on functioning, with the addition of PANSS factors to the same set of independent variables, and SOFAS as the dependent variable. The independent variables were entered into the models based on literature suggesting their association with symptomatic remission [31–38] and functioning [39–42]. The same logistic regression and multiple linear regression analyses were conducted using accommodation as independent variable.

## Results

Description and comparison of socio-demographic and clinical characteristics by living arrangements are presented in Table 1; description and comparison of socio-demographic and clinical characteristics by accommodation are presented in Table 2. The majority had at least secondary education (*n* = 232, 84.06%), mild to moderate severity of psychopathology (PANSS Total: *M* = 58.11, *SD* = 12.88), and moderate (*n* = 91, 37.44%) to serious (*n* = 80, 32.92%) functional impairment.

**Table 1:** Description and comparison of socio-demographic and clinical characteristics by living arrangements

Variables	Mean (SD) or n (%)					Comparisons
	All	Spouse/ Partner	Family	Independent	Rehabilitation	
Living arrangements	276 (100%)	33 (11.96%)	185 (67.03%)	17 (6.16%)	41 (14.86%)	
Housing						*χ*^2^(9) = 312.207, *p* < 0.001
Private	15 (5.43%)	3 (9.09%)	12 (6.49%)	–	–	
Public	180 (65.22%)	24 (72.73%)	150 (81.08%)	6 (35.29%)	–	
Rental	40 (14.49%)	6 (18.18%)	23 (12.43%)	11 (64.71%)	–	
Rehabilitation centres	41 (14.86%)	–	–	–	41 (100%)	
Sex (Male)	152 (55.07%)	14 (42.42%)	105 (56.76%)	10 (58.82%)	23 (56.10%)	*χ*^2^(3) = 2.460, *p* = 0.483
Age, years	40.42 (10.14)	45.85 (8.19)	38.38 (9.76)	46.71 (8.38)	42.66 (11.01)	*F*(3, 272) = 9.267, *p* < 0.001
20–29	46 (16.67%)	0 (0.00%)	38 (82.61%)	0 (0%)	8 (17.39%)	*χ*^2^(12) = 27.805, *p* = 0.006
30–39	86 (31.16%)	7 (8.14%)	67 (77.91%)	3 (3.49%)	9 (10.47%)	
40–49	83 (30.07%)	15 (18.07%)	49 (59.04%)	7 (8.43%)	12 (14.46%)	
50–59	53 (19.20%)	9 (16.98%)	28 (52.83%)	6 (11.32%)	10 (18.87%)	
60–69	8 (2.90%)	2 (25.00%)	3 (37.50%)	1 (12.50%)	2 (25.00%)	
Age of onset of illness, years	23.11 (6.50)	25.79 (7.82)	22.29 (6.10)	23.06 (4.62)	24.66 (7.06)	*H*(3) = 8.481*, p* = 0.037
Ethnicity^1^						*χ*^2^(6) = 11.258, *p* = 0.081
Chinese	233 (84.73%)	28 (12.02%)	163 (69.96%)	13 (5.58%)	29 (12.45%)	
Malay	20 (7.27%)	3 (15.00%)	10 (50.00%)	1 (5.00%)	6 (30.00%)	
Indian	22 (8.00%)	1 (4.55%)	12 (54.55%)	3 (13.64%)	6 (27.27%)	
Education, years	13.17 (3.12)	13.05 (3.43)	13.41 (3.01)	13.02 (3.22)	12.23 (3.21)	*F*(3, 272) = 1.636, *p* = 0.181
Smoking status^2^						*χ*^2^(6) = 9.569, *p* = 0.144
Current smoker	60 (21.98%)	4 (12.12%)	37 (20.22%)	8 (47.06%)	11 (27.50%)	*χ*^2^(3) = 9.148, *p* = 0.027 ^4^
Ex-smoker	36 (13.19%)	5 (15.15%)	26 (14.21%)	1 (5.88%)	4 (10.00%)	
Non-smoker	177 (64.84%)	24 (72.73%)	120 (65.57%)	8 (47.06%)	25 (62.50%)	
Employed	127 (46.01%)	19 (57.58%)	91 (49.19%)	6 (35.29%)	11 (26.83%)	*χ*^2^(3) = 9.388, *p* = 0.025
Physical comorbidity	178 (64.49%)	23 (69.70%)	113 (61.08%)	14 (82.35%)	28 (68.29%)	*χ*^2^(3) = 3.957, *p* = 0.266
Antipsychotic dose*, mg	461.67 (406.25)	314.78 (305.71)	453.95 (400.13)	603.35 (574.38)	556.02 (394.61)	*H*(3) = 11.856, *p* = 0.008
Illness exacerbation	2.98 (1.48)	2.45 (1.15)	2.91 (1.49)	3.76 (1.64)	3.39 (1.37)	*H*(3) = 12.636, *p* = 0.005
SOFAS	55.51 (11.20)	63.61 (12.56)	54.89 (11.31)	51.00 (9.46)	53.95 (7.25)	Brown-Forsythe *F*(3, 74.752) = 7.390, *p* < 0.001
PANSS Total	58.11 (12.88)	50.41 (11.11)	59.08 (13.14)	61.59 (13.83)	58.32 (10.62)	*H*(3) = 14.457, *p* = 0.002
PANSS Positive	8.31 (4.35)	6.13 (3.02)	8.46 (4.43)	9.88 (4.73)	8.66 (4.28)	*H*(3) = 11.630, *p* = 0.009
PANSS Negative	11.28 (4.05)	9.31 (3.11)	11.66 (4.02)	11.35 (4.26)	11.12 (4.42)	*H*(3) = 9.412, *p* = 0.024
PANSS Excitement	4.60 (2.06)	4.31 (1.91)	4.73 (2.14)	4.82 (2.24)	4.15 (1.65)	*H*(3) = 2.625, *p* = 0.453
PANSS Depression	5.60 (2.50)	4.97 (2.09)	5.69 (2.56)	6.29 (3.08)	5.41 (2.21)	*H*(3) = 2.693, *p* = 0.441
PANSS Cognition	4.34 (1.64)	3.81 (1.55)	4.40 (1.67)	4.18 (1.38)	4.54 (1.66)	*H*(3) = 4.341, *p* = 0.227
Symptomatic remission^3^	76 (27.74%)	17 (53.13%)	49 (26.63%)	3 (17.65%)	7 (17.07%)	*χ*^2^(3) = 13.592, *p* = 0.004

*Antipsychotic doses were converted into total daily chlorpromazine equivalents

^1^ Valid percentages were presented; 1 individual with other ethnicity living with spouse excluded

^2^ Valid percentages were presented; 2 missing data observed in people living with parents, 1 missing data in people living in rehabilitation centres

^3^ Valid percentages were presented; 1 missing data observed in people living with spouse, 1 missing data in people living with family

^4^ Association between current smoker vs. ex-smoker/non-smoker and 4 types of living arrangements

SOFAS, Social and Occupational Functioning Scale; PANSS, Positive and Negative Syndrome Scale

**Table 2:** Description and comparison of socio-demographic and clinical characteristics by accommodation

Variables	Mean (SD) or n (%)					Comparisons (Owned vs. Rehabilitation/ Rental accommodation)
	All	Owned accommodation	Rehabilitation/ Rental accommodation	Rental accommodation	Rehabilitation centres	
Accommodation	276 (100%)	195 (70.65%)	81 (29.35%)	40 (14.49%)	41 (14.86%)	
Living arrangements						*χ*^2^(3) = 136.467, *p* < 0.001
Spouse	33 (11.96%)	27 (13.85%)	6 (7.41%)	6 (15.00%)	–	
Family	185 (67.03%)	162 (83.08%)	23 (28.40%)	23 (57.50%)	–	
Independent	17 (6.16%)	6 (3.08%)	11 (13.58%)	11 (27.50%)	–	
Rehabilitation centres	41 (14.86%)	–	41 (50.62%)	–	41 (100.00%)	
Sex (Male)	152 (55.07%)	107 (54.87%)	45 (55.56%)	22 (55.00%)	23 (56.10%)	*χ*^2^(1) = 0.011, *p* = 0.917
Age, years	40.42 (10.14)	39.95 (9.93)	41.56 (10.60)	40.42 (10.19)	42.66 (11.01)	*t*(274) = 1.200, *p* = 0.231
20–29	46 (16.67%)	32 (69.57%)	14 (30.43%)	6 (13.04%)	8 (17.39%)	*χ*^2^ (4) = 3.734, *p* = 0.443
30–39	86 (31.16%)	63 (73.26%)	23 (26.74%)	14 (16.28%)	9 (10.47%)	
40–49	83 (30.07%)	62 (74.40%)	21 (25.30%)	9 (10.84%)	12 (14.46%)	
50–59	53 (19.20%)	32 (60.38%)	21 (39.62%)	11 (20.75%)	10 (18.87%)	
60–69	8 (2.90%)	6 (75.00%)	2 (25.00%)	0 (0.00%)	2 (25.00%)	
Age of onset of illness, years	23.11 (6.50)	22.79 (6.54)	23.85 (6.36)	23.03 (5.52)	24.66 (7.06)	*U* = 6991.5*, z* = −1.503*, p* = 0.133
Ethnicity^1^						*χ*^2^(2) = 8.377, *p* = 0.015
Chinese	233 (84.73%)	173 (74.25%)	60 (25.75%)	31 (13.30%)	29 (12.45%)	
Malay	20 (7.27%)	11 (55.00%)	9 (45.00%)	3 (15.00%)	6 (30.00%)	
Indian	22 (8.00%)	11 (50.00%)	11 (50.00%)	5 (22.73%)	6 (27.27%)	
Education, years	13.17 (3.12)	13.75 (2.99)	11.77 (2.98)	11.29 (2.67)	12.23 (3.21)	*t*(274) = −5.009, *p* < 0.001
Smoking status^2^						*χ*^2^(2) = 19.321, *p* < 0.001
Current smoker	60 (21.98%)	29 (14.95%)	31 (39.24%)	20 (51.28%)	11 (27.50%)	*χ*^2^(1) = 19.319, *p* < 0.001 ^4^
Ex-smoker	36 (13.19%)	28 (14.43%)	8 (10.13%)	4 (10.26%)	4 (10.00%)	*χ*^2^(4) = 26.164, *p* < 0.001 ^5^
Non-smoker	177 (64.84%)	137 (70.62%)	40 (50.63%)	15 (38.46%)	25 (62.50%)	
Employed	127 (46.01%)	95 (48.72%)	32 (39.51%)	21 (52.50%)	11 (26.83%)	*χ*^2^(1) = 1.955, *p* = 0.162
Physical comorbidity	178 (64.49%)	124 (63.59%)	54 (66.67%)	26 (65.00%)	28 (68.29%)	*χ*^2^(1) = 0.237, *p* = 0.627
Antipsychotic dose*, mg	461.67 (406.25)	435.18 (390.66)	525.47 (437.49)	494.15 (480.54)	556.02 (394.61)	*U* = 7000.5*, z* = −1.487*, p* = 0.137
Illness exacerbation	2.98 (1.48)	2.85 (1.47)	3.29 (1.44)	3.20 (1.52)	3.39 (1.37)	*U* = 6256.0*, z* = −2.282*, p* = 0.022
SOFAS	55.51 (11.20)	55.73 (11.45)	54.99 (10.67)	56.08 (13.39)	53.95 (7.25)	*t*(241) = −0.478, *p* = 0.633
PANSS Total	58.11 (12.88)	57.96 (13.43)	58.46 (11.53)	58.60 (12.54)	58.32 (10.62)	*U* = 7467.5*, z* = −0.583*, p* = 0.560
PANSS Positive	8.31 (4.35)	8.31 (4.47)	8.30 (4.10)	7.93 (3.92)	8.66 (4.28)	*U* = 7634.5*, z* = −0.307*, p* = 0.759
PANSS Negative	11.28 (4.05)	11.34 (4.00)	11.16 (4.18)	11.20 (3.98)	11.12 (4.42)	*U* = 8100.0*, z* = − 0.475*, p* = 0.635
PANSS Excitement	4.60 (2.06)	4.59 (2.08)	4.62 (2.00)	5.10 (2.22)	4.15 (1.65)	*U* = 7614.5*, z* = −0.361*, p* = 0.718
PANSS Depression	5.60 (2.50)	5.56 (2.52)	5.70 (2.46)	6.00 (2.68)	5.41 (2.21)	*U* = 7499.0*, z* = −0.542*, p* = 0.588
PANSS Cognition	4.34 (1.64)	4.24 (1.60)	4.57 (1.72)	4.60 (1.79)	4.54 (1.66)	*U* = 7007.0*, z* = −1.381*, p* = 0.167
Symptomatic remission^3^	76 (27.74%)	56 (29.02%)	20 (24.69%)	13 (32.50%)	7 (17.07%)	*χ*^2^(1) = 0.532, *p* = 0.466

*Antipsychotic doses were converted into total daily chlorpromazine equivalents

^1^ Valid percentages were presented; 1 individual with other ethnicity living in rental accommodation excluded

^2^ Valid percentages were presented; 1 missing data observed in each of the three accommodation categories

^3^ Valid percentages were presented; 2 missing data observed in owned accommodation

^4^ Association between current smoker vs. ex-smoker/non-smoker and owned accommodation vs. rental accommodation/rehabilitation centres

^5^ Association between 3 smoking categories and 3 accommodation categories

SOFAS, Social and Occupational Functioning Scale; PANSS, Positive and Negative Syndrome Scale

The majority who lived with a spouse (*n* = 27, 81.82%) or family (*n* = 162, 87.57%) lived in an owned accommodation, while the majority who lived independently lived in a rental accommodation (*n* = 11, 64.71%), *χ*^2^(3) = 136.467, *p* < 0.001.

### Association with socio-demographic variables

#### Living arrangements

Age was significantly different by living arrangements, *F* (3, 272) = 9.267, *p* < 0.001. People living with family were significantly younger than people living with a spouse (*p* < 0.001) and people living independently (*p* = 0.005).

A higher proportion of current smokers was found in people living independently, *χ*^2^(3) = 9.148, *p* = 0.027. However, when ex-smokers and non-smokers were examined separately, the difference in proportions was not statistically significant, *χ*^2^(6) = 9.569, *p* = 0.144.

#### Accommodation

People living in owned accommodation had more years of education (*M* = 13.75, *SD* = 2.99) than people living in rental accommodation (*M* = 11.29, SD = 2.67 *p* < 0.001) and rehabilitation centres (*M* = 12.23, *SD* = 3.21 *p* = 0.010), *F* (2, 273) = − 13.597, *p* < 0.001.

Twenty (51.28%) people living in rental accommodation were smokers, as compared to 11 (27.50%) in rehabilitation centres, and 29 (14.95%) in owned accommodation, *χ*^2^(4) = 26.164, *p* < 0.001.

### Association with clinical variables

#### Living arrangements

Antipsychotic dose, *H* (3) = 11.856, *p* = 0.008, and age of onset of psychotic symptoms, *H* (3) = 8.481, *p* = 0.037, were significantly different by living arrangements. People living in rehabilitation centres (*U* = -59.108, *z* = − 3.169, *p* = 0.009) were taking significantly higher antipsychotic doses than people living with a spouse. Age of onset of psychotic symptoms was not significantly different in pairwise comparisons.

There was a significant difference in illness exacerbation by living arrangements, *H* (3) = 12.636, *p* = 0.005. People living with a spouse had significantly fewer illness exacerbations than people living in rehabilitation centres (*U* = -49.261, *z* = − 2.690, *p* = 0.043), and people living independently (*U* = -69.535, *z* = − 3.026, *p* = 0.015).

#### Accommodation

Significantly fewer illness exacerbations were observed in people living in owned accommodation than people living in rental accommodation/rehabilitation centres (*U* = 6256.00, *z* = − 2.282, *p* = 0.022). The overall difference between the three accommodation categories was marginally significant, *H (2)* = 5.495, *p* = 0.064, although people living in rehabilitation centres (158.08) and rental accommodation (148.75) had higher mean rank than that of people living in owned accommodation (129.75).

### Association with symptoms

#### Living arrangements

Univariate analyses suggested significant differences in distribution of PANSS Total, *H (3*) = 14.457, *p* = 0.002, PANSS Positive, *H* (3) = 11.630, *p* = 0.009, and PANSS Negative, *H* (3) = 9.412, *p* = 0.024, by living arrangements. People living with a spouse had significantly lower PANSS Total than people living with family, *U* = -55.787, *z* = − 3.677, *p* = 0.001, people living independently, *U* = -65.924, *z* = − 2.773, *p* = 0.033, and people living in rehabilitation centres, *U* = -52.795, *z* = − 2.826, *p* = 0.028. People living with a spouse had significantly lower PANSS Positive than people living with family, *U* = -44.714, *z* = − 2.973, *p* = 0.018, people living independently, *U* = -66.828, *z* = − 2.836, *p* = 0.027, and people living in rehabilitation centres, *U* = -51.157, *z* = − 2.762, *p* = 0.034. People living with a spouse had significantly lower PANSS Negative than people living with family, *U* = -45.889, *z* = − 3.033, *p* = 0.015.

A higher proportion of people living with a spouse achieved symptomatic remission (*n* = 17, 53.13%) as compared to all other groups (17.07–26.63%), *χ*^2^(3) = 13.592, *p* = 0.004. In logistic regression, later age of onset, *OR* = 1.054, CI [1.001, 1.109], living with a spouse as opposed to living with family, *OR* = 0.328, CI [0.140, 0.770], and living in rehabilitation centres, *OR* = 0.168, CI [0.051, 0.558], were associated with higher likelihoods of symptomatic remission (Table 3).

**Table 3:** Logistic Regression of living arrangements on PANSS remission status

Independent Variables	Overall Symptomatic Remission	
	Exp(B)	Sig.
Sex (Female)	1.044 (0.584–1.865)	0.886
Age, year	0.979 (0.945–1.014)	0.241
Age of illness onset, year	1.054 (1.001–1.109)	0.046
Ethnicity		
Malay	1.021 (0.302–3.453)	0.973
Indian	1.226 (0.425–3.535)	0.707
Physical comorbidities	0.566 (0.310–1.035)	0.065
Education, year	0.943 (0.855–1.039)	0.234
Antipsychotic dose*, mg	0.999 (0.998–1.000)	0.070
Illness exacerbation	0.941 (0.752–1.177)	0.594
Living arrangements (Reference group: Spouse)		
Family	0.328 (0.140–0.770)	0.010
Independent	0.277 (0.061–1.247)	0.094
Rehabilitation centres	0.168 (0.051–0.558)	0.004

*Antipsychotic doses were converted into total daily chlorpromazine equivalents

#### Accommodation

PANSS Total and all PANSS factors were not significantly different by accommodation (see Table 2).

The proportion of symptomatic remission status was not significantly different by accommodation, *χ*^2^(1) = 0.532, *p* = 0.466. Symptomatic remission was also not significantly associated with accommodation in the logistic regression model.

### Association with functioning

#### Living arrangements

SOFAS was significantly different by living arrangements, Brown-Forsythe *F* (3, 74.752) = 7.390, *p* < 0.001. People living with a spouse had significantly higher SOFAS (*M* = 63.61, *SD* = 12.57) than all other groups (family: *M* = 54.89, *SD* = 11.31, *p* = 0.008; independent: *M* = 51.00, *SD* = 9.46, *p* = 0.004; rehabilitation: *M* = 53.95, *SD* = 7.25, *p* = 0.004). Multiple regression revealed that more years of education (*t* = 2.039, *p* = 0.043), fewer illness exacerbations (*t* = − 2.240, *p* = 0.026), less severe PANSS Negative (*t* = − 6.232, *p* < 0.001), living with a spouse as compared to living with family (*t* = − 2.865, *p* = 0.005), living independently (*t* = − 2.137, *p* = 0.034) and living in rehabilitation centres (*t* = − 2.413, *p* = 0.017), were associated with better functioning (Table 4).

**Table 4:** Multiple regression of living arrangements on SOFAS

	B	SE	t	Sig.
Age, year	−0.009	0.073	−0.119	0.905
Age of illness onset, year	−0.117	0.108	−1.084	0.279
Sex (Female)	−0.286	1.272	−0.225	0.822
Ethnicity				
Malay	0.865	2.280	0.380	0.705
Indian	3.304	2.166	1.526	0.129
Education, year	0.404	0.198	2.039	0.043
Physical comorbidity	−1.827	1.331	−1.372	0.171
Antipsychotic dose*, mg	−0.003	0.002	−1.729	0.085
Illness exacerbation	−1.096	0.489	−2.240	0.026
PANSS Positive	−0.271	0.171	−1.583	0.115
PANSS Negative	−1.058	0.170	−6.232	< 0.001
PANSS Excitement	−0.295	0.323	−0.914	0.361
PANSS Depression	−0.214	0.281	−0.762	0.447
PANSS Cognition	−0.285	0.429	−0.664	0.507
Living arrangements (Reference group: Spouse)				
Family	−5.980	2.088	−2.865	0.005
Independent	−6.740	3.154	−2.137	0.034
Rehabilitation centres	−5.980	2.478	−2.413	0.017

*Antipsychotic doses were converted into total daily chlorpromazine equivalents

PANSS, Positive and Negative Syndrome Scale

Model summary: *F* (17,221) = 7.732, *p* < 0.001, *R*^2^ = 0.373, Adjusted *R*^2^ = 0.325

A higher proportion of people living with a spouse was employed (*n* = 19, 57.58%) as compared to all other groups (26.83%–49.19%), *χ*^2^(3) = 9.388, *p* = 0.025.

#### Accommodation

Functioning of people living in rental accommodation/rehabilitation centres compared to people living in an owned accommodation was not significantly different, *t* (241) = − 0.478, *p* = 0.633. Similarly, accommodation was not significantly associated with SOFAS in the multiple regression model.

Fewer people living in rental accommodation/rehabilitation centres were employed (*n* = 32, 39.51%), as compared to people living in owned accommodation (*n* = 95, 48.72%), but the differences did not attain statistical significance, *χ*^2^(1) = 1.955, *p* = 0.162. A closer examination found that fewer people living in rehabilitation centres (*n* = 11, 26.83%) were employed, as compared to 21 (52.50%) in rental accommodation and 95 (48.72%) in owned accommodation, *χ*^2^(2) = 7.326, *p* = 0.026.

## Discussion

This study compared socio-demographic, clinical and functional characteristics of people in different living arrangements and accommodation, and provided further evidence on the unique associations of living arrangements with symptomatic remission and functioning. Living arrangements were associated with positive and negative symptoms, symptomatic remission and functioning in people with schizophrenia. People living with a spouse had a higher likelihood to have achieved symptomatic remission as compared to people living with family and in rehabilitation centres, and fewer illness exacerbations as compared to people living independently and in rehabilitation centres. They also had significantly better functioning than all other groups. A higher proportion of people living with a spouse were employed. Although people living in owned accommodation had more years of education and fewer illness exacerbations than people living in rental accommodation and rehabilitation centres, no significant differences were found on their PANSS factor scores, symptomatic remission status, and functioning. A lower proportion of people living in rehabilitation centres were employed as compared to people living in owned or rental accommodation. Smoking was related to both living arrangements and accommodation.

Approximately 79% of participants lived with family or spouse, similar to the percentage reported in Japan [43] and higher than the percentage reported in the United States [10]. In Oshima & Kuno (2006), a higher percentage of people aged 40–49 than people aged 30–39 lived independently [43]. In our study, the percentage of people living with family declined with age, while the percentage of people living independently increased with age, probably due to the unavailability of family as they age. People aged 20–29 were either living with parents or in rehabilitation centres, consistent with the findings on older age of nest-leaving in youth in Asia [19]. Accommodation of participants in our study is generally a reflection of the types of residential occupancy in Singapore, in which the majority of residents live in public housing (78.97% in 2017 to 80.43% in 2014) and approximately 20% (19.27% in 2014 to 20.79% in 2017) live in private housing [44].

People living with a spouse had significantly lower PANSS Positive than all other groups, lower PANSS Negative than people living with family, and a higher likelihood to have achieved symptomatic remission, while people living independently had the highest mean PANSS Total score and PANSS Positive. These findings suggested that social ties in living arrangements may be relevant in symptom management. Social ties are beneficial to mental health, through social integration and positive social influences, and by modulating stress reaction and making support more accessible [45]. Social interaction would help patients with reality testing and to evaluate their own behaviors by comparing it with others’ behaviors [5]. Contrarily, independent living was associated with social isolation [46], and social isolation was associated with poorer mental health [47]. Social isolation, loneliness and the lack of communication were also associated with paranoia and hallucination [48, 49]. The lack of social integration might explain the more severe positive symptoms found in people living independently in our study. Conversely, people living with a spouse were more integrated socially and had lower psychotic symptoms [3]. It is possible that living with a spouse provided an environment that could enhance social network, which also provided material and psychological support in recovery process [5, 6]. The findings that people living with their partner maintained relatively intensive interaction with family and extra-family members and had a confidant more often than people living with parents or independently [4] suggested that people living with a spouse may have lower Asociality. This is consistent with the finding which suggested that social network was inversely correlated with negative symptoms [3]. Alternatively, it is possible that people with better managed positive symptoms and lower Asociality were more capable to engage in reciprocal social relationships and therefore got married. In addition, positive symptoms such as paranoia may cause social avoidance. Increase in positive symptoms was associated with decrease in reciprocal social relationship, subjective satisfaction with social relationship and increase in loneliness [50].

Family environment could be either a protective or predisposing factor on outcomes in people with schizophrenia. A study suggested that adoptees in healthy adoptive family environment had little serious psychopathology, while adoptees in disturbed adoptive family environment had higher serious psychopathology, regardless of whether their biological mother had schizophrenia [51]. Although family support may be readily available for people living with family, family emotional environment, interaction, dynamics, caregivers’ coping capabilities, and amount of contact may be relevant to symptoms management [52], which may be the underlying factors of the higher PANSS Positive and PANSS Negative in people living with family as compared to people living with spouse found in our study. Alternatively, it’s possible that people were living with their family because they did not manage to find a partner due to their higher positive and negative symptoms, as suggested by the marriage selection mechanism [53].

We found that people living in rehabilitation centres had significantly higher positive symptoms, more illness exacerbations, and were receiving significantly higher doses of antipsychotic than people living with a spouse. They had fewer years of education than people living in owned accommodation, and the proportion of them being employed was also lower than those living in owned and rental accommodation, respectively. This is in line with previous literature that people living in residential facilities were more likely to be less educated and unemployed [24, 46]. Tsai et al. (2011) showed that schizophrenia patients who remained living in an institution for 12 months had more severe positive symptoms than people in other living arrangements [10]. In the local setting, residential rehabilitation centres support patients in their recovery stage, therefore it is possible that people living in rehabilitation centres had more severe positive symptoms. The latter could in turn be associated with prescription of higher doses of antipsychotics and lower likelihood to be employed. On the other hand, living in residential facilities contributed to physical alienation in addition to alienation resulting from psychotic experiences [54]. Communal living with people having mental health issues could be stressful [55] and may not be helpful to develop a wider social network and to be included in the mainstream social network. A higher number of activities completed in a social inclusion programme were associated with lower symptoms, less social and occupational problems, and better functioning in people with psychosis [56]. The opportunity to be more socially included might be beneficial to recovery, which unfortunately is intertwined and complicated by the patients’ clinical states and readiness to engage in social and occupational activities.

Living with a spouse was associated with better functioning and a higher likelihood to be employed. Previous studies also showed that married patients with schizophrenia had better functioning, were more often employed, and did more useful work than non-married patients [3, 57]. Having stable partnership may be advantageous to outcome in people with schizophrenia [58]. Nevertheless, the better outcomes in people living with a spouse could be due to their better premorbid functioning and better prognosis [59]. Being able to maintain their married status could be the results of personal characteristics such as lower severity of illness and lower Asociality, which are also the significant factors of better functioning [60]. Better functioning may help people to maintain their married status too.

Our results showed that PANSS Positive, PANSS Negative, symptomatic remission and functioning were associated with living arrangements, but not types of accommodation. This suggested that people whom the participants lived with may be more important. The results were consistent with a previous study that severity of positive symptoms did not differ significantly between people living in boarding house and people living in owned or rental accommodation [61]. However, our results were contrary to findings in previous studies which suggested that residential independence was associated with lower negative symptoms [62–64] and better functioning [17, 61, 62]. It’s suggested that people with schizophrenia living in owned or rental accommodation had better functioning than people living in boarding house [61]. The interaction of factors that may impact functioning such as adequacy of accommodation [12], available social support and meaningful activities in the environment [61, 65] might be the underlying factors.

Types of accommodation may not imply residential independence in our context. The choice of accommodation might depend on the personal resources and interpersonal network the participant has and may not always be an autonomous decision. A study also suggested that the majority of people with chronic schizophrenia had to rely on the “natural living arrangements” such as family settings due to the shortage of alternative accommodation and personal economic situation [66]. Additionally, multi-generational co-residence is common in Asia, including Singapore [20, 67]. The proportion of young people leaving home was lower and age leaving parental homes was higher in Asian countries [19]. Independent living as a transition to adulthood may be the expectation in the West but not necessarily the expectation in the East, which may explain the finding of better functioning in people living independently in the United States [10] but not in ours. The wider socio-cultural environment should be understood to better comprehend the different findings on living arrangements.

People living independently had the highest illness exacerbations while people living with a spouse had the lowest. This is consistent with previous research that people living independently were most often hospitalized while people living with a spouse were least often hospitalized [3], but contrary to Tsai et al.’s (2011) where they reported patients living alone independently were less likely to be re-hospitalized. Social support may be relevant—hospitalization due to a lack of social support was more often the cause than illness exacerbation in some chronic psychiatric patients [68]. We also found that people living in owned accommodation had significantly fewer illness exacerbations than people living in rental accommodation and rehabilitation centres, consistent with the findings that people with schizophrenia living in their own home had a lower likelihood of re-hospitalization than people living in boarding house [18] and number of re-hospitalizations were positively associated with number of changes in living arrangements [69]. The stability of accommodation and the resulting stability of interpersonal environment, sense of security, belonging and familiarity may be protective factors [11].

The highest percentage of smokers and lowest percentage of ex-smokers were found in people living independently. Living arrangements may have protective utility, as people living with family, spouse and in rehabilitation centres may be discouraged from smoking or face more restriction in smoking. Additionally, increased tobacco use was associated with social isolation [47], which is more likely in people living independently. Furthermore, a higher proportion of people living in rental accommodation were smokers, consistent with the literature that smoking is more prevalent in the socio-economic disadvantaged [70] and people with lower income [71]. Lee et al. (2019) showed that residents with schizophrenia in homes smoked more cigarettes, suggesting living arrangements may influence smoking behavior [72].

This study has some limitations. Firstly, the cross-sectional nature of the study did not allow causal relationship to be drawn, therefore the inability to identify whether living arrangements were the consequence of symptoms and functioning, or served as a protective mechanism that encouraged healthy life styles, and promoted symptomatic and functional recovery. Secondly, it is important to note that the generalizability of these results is limited to the context of the study and characteristics of the sample. Singapore is urbanised with good accessibility to healthcare service and necessities in daily life. Also, all participants were community dwelling outpatients and most of them had mild to moderate severity of symptoms. Further, factors associated with symptoms and functioning within living arrangements such as interpersonal dynamics, emotional environment, extent of support and amount of contact were not investigated, limiting possible insights that could be drawn.

## Conclusion and recommendation

To conclude, living with a spouse was associated with better outcomes, while people living independently had the highest symptom severity and lowest functioning. Types of accommodation was not associated with symptoms and functioning. The results suggested that living arrangements are important in the care for outpatients with schizophrenia and more attention should be given to patients living independently in the community. Programmes to enhance social inclusion might be beneficial for people living in rehabilitation centres. In addition, family and partners of outpatients with schizophrenia should be provided with education and support, to ease their caregiving burden and to maintain a supportive environment for recovery. Family assessments may be useful to identify caregivers who need support, to invite them to participate in family programmes or caregiver alliances.

## Data Availability

The participants of this study did not agree for their data to be shared publicly, so supporting data is not publicly available. Further enquiries could be directed to either the corresponding author or to imhresearch@imh.com.sg.

## Abbreviations

ANOVA: One-way analysis of variance
PANSS: Positive and Negative Syndrome Scale
SCID-I/P: DSM-IV-TR Axis I Disorder-Patient Edition
SOFAS: Social and Occupational Functioning Scale
